# Supplementary material for: Effects of perinatal mobile apps for couples on psychosocial and parenting outcomes: A systematic review and meta-analysis
Source: PLOS Ment Health. 2025 Oct 8;2(10):e0000432. doi: 10.1371/journal.pmen.0000432 (PMC12798352; doi:10.1371/journal.pmen.0000432)
Supplement: S2 Appendix — (DOCX) [file pmen.0000432.s003.docx]

**S2 Appendix. R script for extracting data from a published figure**

The following R script was used to extract numerical data (means, standard deviations, and confidence intervals) from **Figure 2.1** of:

Garfield CF, Lee YS, Kim HN, Rutsohn J, Kahn JY, Mustanski B, et al. Supporting parents of premature infants transitioning from the NICU to home: A pilot randomized control trial of a smartphone application. Internet Interventions. 2016;4:131–137. doi:10.1016/j.invent.2016.05.004

The figure itself is not included here due to copyright restrictions.
Users who wish to replicate the extraction process should obtain the original article and save the figure as a PNG image before running the script. The code can be adapted to other figures by adjusting the xlim and ylim parameters.

# ==========================================================

# S4 Appendix - R script for extracting data from a figure

# Target: Figure 2.1 from Garfield et al., 2016

# R version: 4.3.2

# ==========================================================

# --- 1. Load required packages ---

# Install 'png' if not already installed: install.packages("png")

library(png) # To read PNG images

# --- 2. Load the figure (user must provide their own copy) ---

# Save the figure as a PNG file and update the path below

image_path <- "figure_2.1_garfield2016.png"

figure <- readPNG(image_path)

# --- 3. Prepare the plotting area ---

par(mar = c(0, 0, 0, 0)) # No margins

# Create an empty plot with scales matching the original figure

# Adjust ylim/xlim to match the figure's axis ranges

plot(

NA, xlim = c(0, 1), ylim = c(59, 91),

type = "n", xlab = "", ylab = "",

xaxt = "n", yaxt = "n", bty = "n"

)

# Overlay the figure on the plotting area

rasterImage(figure, 0, 59, 1, 91)

# --- 4. Extract data by clicking on points ---

# Click each point to record its Y-value; press ESC to stop

extract_data <- function() {

repeat {

point <- locator(1)

if (is.null(point)) break

cat("Point height (Y-axis):", point$y, "\n")

}

}

# Run the interactive extraction

extract_data()

**Notes:**

Replace "figure_2.1_garfield2016.png" with the actual path to the saved figure.

The axis limits (xlim and ylim) must match those of the original chart.

This method records the clicked coordinates; additional transformations may be needed to convert them to reported units.
