# Supplementary material for: Effects of perinatal mobile apps for couples on psychosocial and parenting outcomes: A systematic review and meta-analysis
Source: PLOS Ment Health. 2025 Oct 8;2(10):e0000432. doi: 10.1371/journal.pmen.0000432 (PMC12798352; doi:10.1371/journal.pmen.0000432)
Supplement: S1 File — (DOCX) [file pmen.0000432.s004.docx]

**PROSPERO**

International prospective register of systematic reviews

Effects of perinatal mobile applications (Apps) on the psychosocial and parenting outcomes of fathers: a systematic review of randomized clinical trials.

*Alvaro Taype-Rondan, Evelyn M. Asencios-Falcón, Jean Pierre López-Mesia, Liz Mendoza- Aucaruri, Luis Ttito-Paricahua, Marlene Magallanes-Corimanya, Alicia Lopez-Gomero*

To enable PROSPERO to focus on COVID-19 submissions, this registration record has undergone basic automated checks for eligibility and is published exactly as submitted. PROSPERO has never provided peer review, and usual checking by the PROSPERO team does not endorse content. Therefore, automatically published records should be treated as any other PROSPERO registration. Further detail is provided here.

###### Citation

Alvaro Taype-Rondan, Evelyn M. Asencios-Falcón, Jean Pierre López-Mesia, Liz Mendoza-

Aucaruri, Luis Ttito-Paricahua, Marlene Magallanes-Corimanya, Alicia Lopez-Gomero. Effects of perinatal mobile applications (Apps) on the psychosocial and parenting outcomes of fathers: a systematic review of randomized clinical trials.. PROSPERO 2024 Available from http[s://www.crd.york.ac.uk/PROSPERO/view/CRD42024578397](http://www.crd.york.ac.uk/PROSPERO/view/CRD42024578397)

## REVIEW TITLE AND BASIC DETAILS

Review title

Effects of perinatal mobile applications (Apps) on the psychosocial and parenting outcomes of fathers: a systematic review of randomized clinical trials.

#### Original language title

Effects of perinatal mobile applications (Apps) on the psychosocial and parenting outcomes of fathers: a systematic review of randomized clinical trials.

###### Review objectives

Which are the effects of perinatal mobile applications (Apps) on the psychosocial and parenting outcomes of couples and fathers?

Keywords: Mental Health, MeSH: Mobile Applications, Parenting, Social Welfare

## SEARCHING AND SCREENING

Searches

For the literature search, we will use the following electronic databases: PubMed, Embase, and CENTRAL. Furthermore, we will explore the references of the included studies and previous systematic reviews to identify potential studies for inclusion. No restrictions will be placed based on language or publication date.

##### Study design

We will include randomized clinical trials that evaluate the effects of perinatal mobile applications (Apps) designed to improve mental health outcomes (e.g., stress, anxiety, depression), social well- being (e.g., social support), and parenting support (e.g., breastfeeding, paternal attachment, self- efficacy) among parents, compared to standard care. We will accept original articles published in peer-reviewed scientific journals.

## ELIGIBILITY CRITERIA

Condition or domain being studied

Mental health Social well-being Parenting

Population

Couples and fathers in the perinatal period (pregnancy and peripartum) with living children.

##### Intervention(s) or exposure(s)

Mobile applications used in the perinatal stage, aimed at couples and/or fathers with live newborns, designed to improve mental health, social outcomes, and parenting. These applications may be supplemented with other interventions (virtual or in-person), but the primary intervention must be through the mobile application

Comparator(s) or control(s)

No mobile App

The comparison group will be any type of control group that does not use mobile applications to provide mental health, social outcomes, and parenting support to couples or parents during the perinatal period.

Context

Mobile applications (Apps) are increasingly utilized for preventive or supplementary health care activities due to their significant impact on improving the mental health and social well-being of parents, particularly during the first year of life. However, existing literature predominantly focuses on mothers. Consequently, there is an urgent need to synthesize the available evidence to

elucidate the effects of these applications on the mental and social health of couples (both parents) and fathers. This synthesis is crucial for guiding public health initiatives.

# OUTCOMES TO BE ANALYSED

Main outcomes

- Mental health outcomes (such as anxiety, depression, stress) for parents during the first year of life
- Social well-being outcomes (such as social support, attachment)
- Parenting support (e.g., breastfeeding, paternal attachment, parent-child bonding, parental self- efficacy)

*Measures of* effect

When possible, meta-analyses will be conducted using data from multiple studies. Random effects models and the Dersimonian and Laird method will be utilized for analysis. Results will be expressed as relative risks (RR) / odds ratios (OR) / mean differences (MD), along with their corresponding 95% confidence intervals (95% CI) for dichotomous and continuous outcomes, respectively.

###### Additional outcomes

Other mental or social health outcomes, such as feelings of sadness, the blues, participation in peer support groups, and others.

*Measures of effect*

When possible, meta-analyses will be conducted using data from multiple studies. Random effects models and the Dersimonian and Laird method will be utilized for analysis. Results will be expressed as relative risks (RR) / odds ratios (OR) / mean differences (MD), along with their corresponding 95% confidence intervals (95% CI) for dichotomous and continuous outcomes, respectively.

## DATA COLLECTION PROCESS

###### Data extraction (selection and coding)

One of the authors will download all found references to Rayyan QCRI web application (https://rayyan.qcri.org) and eliminate any duplicate articles. Following this, two authors will individually review the titles and abstracts to identify potential studies for inclusion. Subsequently, the authors will independently assess the full texts to ascertain eligibility. Any discrepancies will be resolved through discussion with another reviewer. Subsequently, two authors will independently compile relevant data from the included articles into a Microsoft Excel spreadsheet.

Risk of bias (quality) assessment

Independently, two reviewers will analyze the risk of bias (RoB) of eligible studies. The RoB of randomized controlled trials will be assessed using the revised Cochrane risk-of-bias tool for randomized trials.

# PLANNED DATA SYNTHESIS

###### Strategy for data synthesis

If appropriate, random effects meta-regressions will be conducted to explore the linear relationship between covariates at the study level and effect size.

Heterogeneity will be assessed using a x“ test (Cochran's Q statistic), and the extent of heterogeneity will be quantified using the I* statistic. An 12 value less than 40%, between 40% and

80%, and greater than 80% will indicate low, moderate, and high heterogeneity, respectively. Moreover, we will attempt to evaluate publication bias through funnel plots and Begg's rank test if feasible. The RevMan software will be employed for conducting the meta-analyses.

###### Analysis of subgroups or subsets

Population: couples vs father; heterosexual couples versus homosexual couples

Intervention: characteristics of Apps intervention (such type of Apps, time of use, period of use, evaluation period, etc.)

Control: active control vs usual care

## REVIEW AFFILIATION, FUNDING AND PEER REVIEW

Review team members

- - Alvaro Taype-Rondan, EviSalud - Eviencias en Salud, Perú
  - Evelyn M. Asencios-Falcón, Universidad de San Martin de Porres, Perú
  - Jean Pierre López-Mesia, Facultad de Medicina Humana, Universidad Nacional de la Amazonia Peruana
  - Liz Mendoza-Aucaruri, Carrera de Medicina Humana, Universidad Científica del Sur, Lima, Perú.
  - Luis Ttito-Paricahua, Universidad Científica del Sur, Lima, Perú.
  - Marlene Magallanes-Corimanya, Universidad de San Martin de Porres, Perú
  - Alicia Lopez-Gomero, Universidad Continental, Lima, Perú

#### Review affiliation

EviSalud - Evidencias en Salud, Lima, Perú

###### Funding source

Self-funded

<Vdiv>

##### Named contact

Alvaro Taype-Rondán. [alvaro.taype.r@gmaiI.com](mailto:alvaro.taype.r@gmaiI.com)

## TIMELINE OF THE REVIEW

Review timeline

Start date: 04 August 2024. End date: 04 February 2025

Date of first submission to PROSPERO

12 August 2024

Date of registration in PROSPERO

24 August 2024

### CURRENT REVIEW STAGE

Publication of review results

The intention is not to publish the review once completed.

Stage of the review at this submission

Review stage Pilot work

Formal searching/study identification

Screening search results against inclusion criteria Data extraction or receipt of IP

Risk of bias/quality assessment Data synthesis

Review status

The review is currently planned or ongoing.

### ADDITIONAL INFORMATION

PROSPERO version history

- - Version 1.1 published on 24 Aug 2024
  - Version 1.0 published on 24 Aug 2024

Review conflict of interest

None known

Country Peru

Started Completed

Medical Subject Headings

Fathers; Female; Humans; Male; Mobile Applications; Parenting; Parturition; Pregnancy; Randomized Controlled Trials as Topic

#### Details of any existing review of the same topic by the same authors

No

###### Disclaimer

The content of this record displays the information provided by the review team. PROSPERO does not peer review registration records or endorse their content.

PROSPERO accepts and posts the information provided in good faith; responsibility for record content rests with the review team. The owner of this record has affirmed that the information provided is truthful and that they understand that deliberate provision of inaccurate information may be construed as scientific misconduct.

PROSPERO does not accept any liability for the content provided in this record or for its use. Readers use the information provided in this record at their own risk.

Any enquiries about the record should be referred to the named review contact
