# Supplementary material for: Effects of perinatal mobile apps for couples on psychosocial and parenting outcomes: A systematic review and meta-analysis
Source: PLOS Ment Health. 2025 Oct 8;2(10):e0000432. doi: 10.1371/journal.pmen.0000432 (PMC12798352; doi:10.1371/journal.pmen.0000432)
Supplement: S1 Table — (DOCX) [file pmen.0000432.s005.docx]

**S1 Table. Search strategy.**

Date of search: 21 of November 2024

| Database | Search strategy | Results |
| --- | --- | --- |
| Pubmed / MEDLINE | #1 ("Parents"[Mesh] OR "Fathers"[Mesh] OR "Couples Therapy"[Mesh] OR "Pregnant Women"[Mesh] OR paren*[TIAB] OR father*[TIAB] OR couple[TIAB] OR couples[TIAB] OR partner*[TIAB]) AND ("Infant, Newborn"[Mesh] OR "Perinatal Care"[Mesh] OR "Peripartum Period"[Mesh] OR newborn[TIAB] OR perinatal[TIAB] OR peri-natal[TIAB] OR neonat*[TIAB] OR postpartum[TIAB] OR post-partum[TIAB] OR antenatal[TIAB] OR ante-natal[TIAB] OR baby[TIAB] OR pregnan*[TIAB] OR birth*[TIAB])  #2 "Telemedicine"[Mesh] OR "Cell Phone"[Mesh] OR mhealth[TIAB] OR m-health[TIAB] OR smartphone*[TIAB] OR ehealth[TIAB] OR e-health[TIAB] OR mobile*[TIAB] OR iphone[TIAB] OR ipad[TIAB] OR application*[TIAB] OR app[TIAB] OR apps[TIAB] OR telehealth[TIAB] OR tele-health[TIAB] OR telemedicine[TIAB] OR tele-medicine[TIAB] OR telephone[TIAB] OR cellphone[TIAB] OR cell-phone[TIAB]  #3 "Depression"[Mesh] OR "Anxiety"[Mesh] OR "Depressive Disorder"[Mesh] OR "Social Support"[Mesh] OR depressi*[TIAB] OR anxiety[TIAB] OR anxious[TIAB] OR bonding[TIAB] OR mental[TIAB] OR psycholog*[TIAB] OR "social support"[TIAB] OR satisfaction[TIAB] OR self-efficacy[TIAB] OR preparedness[TIAB] OR “peer support”[TIAB]  #4 (clinical[TIAB] AND trial[TIAB]) OR "Clinical Trials as Topic"[Mesh] OR "clinical trial"[PT] OR random*[TIAB]  #1 AND #2 AND #3 AND #4 | 437 |
| CENTRAL | #1 [mh Parents] OR [mh Fathers] OR [mh "Pregnant Women"] OR [mh "Couples Therapy"] OR paren*:ti,ab,kw OR father*:ti,ab,kw OR couple:ti,ab,kw OR couples:ti,ab,kw OR partner*:ti,ab,kw) AND ([mh "Infant, Newborn"] OR [mh "Perinatal Care"] OR [mh "Peripartum Period"] OR newborn:ti,ab OR perinatal:ti,ab OR peri-natal:ti,ab OR neonat*:ti,ab OR postpartum:ti,ab OR post-partum:ti,ab OR antenatal:ti,ab OR ante-natal:ti,ab OR baby:ti,ab OR pregnan*:ti,ab OR birth*:ti,ab  #2 [mh Telemedicine] OR [mh "Cell Phone"] OR mhealth:ti,ab OR m-health:ti,ab,kw OR smartphone:ti,ab,kw OR ehealth:ti,ab OR e-health:ti,ab OR mobile*:ti,ab OR iphone:ti,ab OR ipad:ti,ab OR application*:ti,ab OR app:ti,ab OR apps:ti,ab,kw OR telehealth:ti,ab OR tele-health:ti,ab OR telemedicine:ti,ab OR tele-medicine:ti,ab OR telephone:ti,ab,kw OR cellphone:ti,ab,kw OR cell-phone:ti,ab,kw  #3 ([mh Depression] OR [mh Anxiety] OR [mh "Depressive Disorder"] OR [mh "Mental Health"] OR depressi*:ti,ab OR anxiety:ti,ab OR anxious:ti,ab OR bonding:ti,ab OR mental:ti,ab OR psycholog*:ti,ab OR "social support":ti,ab,kw OR satisfaction:ti,ab OR self-efficacy:ti,ab OR preparedness:ti,ab OR "peer support":ti,ab,kw)  Filter: trials    #1 AND #2 AND #3 | 992 |
| Embase | #1 (Parent/exp OR Father/exp OR 'Couples Therapy'/exp OR 'Pregnant Woman'/exp OR paren*:ti,ab OR father*:ti,ab OR couple:ti,ab OR couples:ti,ab OR partner*:ti,ab) AND (newborn/exp OR 'Perinatal Care'/exp OR newborn:ti,ab OR perinatal:ti,ab OR peri-natal:ti,ab OR neonat*:ti,ab OR postpartum:ti,ab OR post-partum:ti,ab OR antenatal:ti,ab OR ante-natal:ti,ab OR baby:ti,ab OR pregnan*:ti,ab OR birth*:ti,ab)  #2 Telemedicine/exp OR 'mobile phone'/exp OR mhealth:ti,ab OR m-health:ti,ab OR smartphone*:ti,ab OR ehealth:ti,ab OR e-health:ti,ab OR mobile*:ti,ab OR iphone:ti,ab OR ipad:ti,ab OR application*:ti,ab OR app:ti,ab OR apps:ti,ab OR telehealth:ti,ab OR tele-health:ti,ab OR telemedicine:ti,ab OR tele-medicine:ti,ab OR telephone:ti,ab OR cell-phone:ti,ab OR cellphone:ti,ab  #3 Depression/exp OR Anxiety/exp OR depression/exp OR 'social support'/exp OR depressi*:ti,ab OR anxiety:ti,ab OR anxious:ti,ab OR bonding:ti,ab OR mental:ti,ab OR psycholog*:ti,ab OR 'social support':ti,ab OR satisfaction:ti,ab OR self-efficacy:ti,ab OR preparedness:ti,ab OR “peer support”:ti,ab  #4 'randomized controlled trial'/exp OR 'controlled clinical trial'/de OR random*:ti,ab OR 'randomization'/de OR 'intermethod comparison'/exp OR 'intermethod comparison' OR placebo:ti,ab OR compare:ti OR compared:ti OR comparison:ti OR ((evaluated:ab OR evaluate:ab OR evaluating:ab OR assessed:ab OR assess:ab) AND (compare:ab OR compared:ab OR comparing:ab OR comparison:ab)) OR ((open NEXT/1 label):ti,ab) OR (((double OR single OR doubly OR singly) NEXT/1 (blind OR blinded OR blindly)):ti,ab) OR 'double blind procedure'/de OR ((parallel NEXT/1 group*):ti,ab) OR crossover:ti,ab OR 'cross over':ti,ab OR (((assign* OR match OR matched OR allocation) NEAR/6 (alternate OR group OR groups OR intervention OR interventions OR patient OR patients OR subject OR subjects OR participant OR participants)):ti,ab) OR assigned:ti,ab OR allocated:ti,ab OR ((controlled NEAR/8 (study OR design OR trial)):ti,ab) OR volunteer:ti,ab OR volunteers:ti,ab OR 'human experiment'/de OR trial:ti) NOT (((random* NEXT/1 sampl* NEAR/8 ('cross section*' OR questionnaire* OR survey OR surveys OR database OR databases)):ti,ab) NOT ('comparative study'/de OR 'controlled study'/de OR 'randomised controlled':ti,ab OR 'randomized controlled':ti,ab OR 'randomly assigned':ti,ab)) NOT ('cross-sectional study'/de NOT ('randomized controlled trial'/de OR 'controlled clinical study'/de OR 'controlled study'/de OR 'randomised controlled':ti,ab OR 'randomized controlled':ti,ab OR 'control group':ti,ab OR 'control groups':ti,ab)) NOT ('case control*':ti,ab AND random*:ti,ab NOT ('randomized controlled trial'/de OR 'randomised controlled':ti,ab OR 'randomized controlled':ti,ab)) NOT ('systematic review':ti NOT (trial:ti OR study:ti)) NOT (nonrandom*:ti,ab NOT random*:ti,ab) NOT 'random field*':ti,ab NOT (review:ab AND review:it NOT trial:ti) NOT ('we searched':ab AND (review:ti OR review:it)) NOT 'update review':ab NOT (databases NEAR/5 searched):ab NOT ('cochrane database of systematic reviews'/jt OR 'cochrane database of systematic reviews (online)'/jt  #1 AND #2 AND #3 AND #4 | 848 |
| Scopus | #1 TITLE-ABS-KEY(pregnant) OR TITLE-ABS-KEY(paren*) OR TITLE-ABS-KEY(father*) OR TITLE-ABS-KEY(couple) OR TITLE-ABS-KEY(couples) OR TITLE-ABS-KEY(partner*)) AND (TITLE-ABS-KEY(newborn) OR TITLE-ABS-KEY(perinatal) OR TITLE-ABS-KEY(peri-natal) OR TITLE-ABS-KEY(neonat*) OR TITLE-ABS-KEY(postpartum) OR TITLE-ABS-KEY(post-partum) OR TITLE-ABS-KEY(antenatal) OR TITLE-ABS-KEY(ante-natal) OR TITLE-ABS-KEY(baby) OR TITLE-ABS-KEY(pregnan*) OR TITLE-ABS-KEY(birth*)  #2 TITLE-ABS-KEY(Telemedicine) OR TITLE-ABS-KEY(mhealth) OR TITLE-ABS-KEY(m-health) OR TITLE-ABS-KEY(smartphone*) OR TITLE-ABS-KEY(ehealth) OR TITLE-ABS-KEY(e-health) OR TITLE-ABS-KEY(mobile*) OR TITLE-ABS-KEY(iphone) OR TITLE-ABS-KEY(ipad) OR TITLE-ABS-KEY(application*) OR TITLE-ABS-KEY(app) OR TITLE-ABS-KEY(apps) OR TITLE-ABS-KEY(telehealth) OR TITLE-ABS-KEY(tele-health) OR TITLE-ABS-KEY(telemedicine) OR TITLE-ABS-KEY(tele-medicine) OR TITLE-ABS-KEY(telephone) OR TITLE-ABS-KEY(cell-phone) OR TITLE-ABS-KEY(cellphone)  #3 TITLE-ABS-KEY(depressi*) OR TITLE-ABS-KEY(anxiety) OR TITLE-ABS-KEY(anxious) OR TITLE-ABS-KEY(bonding) OR TITLE-ABS-KEY(mental) OR TITLE-ABS-KEY(psycholog*) OR TITLE-ABS-KEY("social support") OR TITLE-ABS-KEY(satisfaction) OR TITLE-ABS-KEY(self-efficacy) OR TITLE-ABS-KEY(preparedness) OR TITLE-ABS-KEY(“peer support”  #4 (TITLE-ABS-KEY(clinical) AND TITLE-ABS-KEY(trial)) OR TITLE-ABS-KEY(random*)  #1 AND #2 AND #3 AND #4 | 1070 |
| Clinicaltrials.gov | #1 Telemedicine* OR Cell Phone* OR m-health* OR smartphone* OR mobile* OR iphone OR ipad OR application* OR app OR cell-phone* OR app*  #2 Paren* OR Father* OR Couple* OR Perinatal* Care OR Peripartum* Period OR peri-natal* OR postpartum* OR antenatal* OR pregnan* OR birth*    Filter: Completed  #1 AND #2 | 765 |
