# Supplementary material for: Effects of perinatal mobile apps for couples on psychosocial and parenting outcomes: A systematic review and meta-analysis
Source: PLOS Ment Health. 2025 Oct 8;2(10):e0000432. doi: 10.1371/journal.pmen.0000432 (PMC12798352; doi:10.1371/journal.pmen.0000432)
Supplement: S2 Table — (DOCX) [file pmen.0000432.s006.docx]

**S2 Table. Excluded studies reviewed at full text stage.**

| **Author (year)** | **Title** | **Exclusion reasons** |
| --- | --- | --- |
| Uccellini  (2022) | 1000 Days: The “WeCare Generation” Program—The Ultimate Model for Improving Human Mental Health and Economics: The Study Protocol | Different intervention |
| Henshaw  (2024) | A randomized controlled trial of the Happy Healthy Loved personalized text-message program for new parent couples: impact on breastfeeding self-efficacy and mood. | Different intervention |
| J Fisher (2011) | What were we thinking! An innovative psycho-educational program to prevent common postpartum mental disorders in women.The Marcé International Society International Biennial General Scientific Meeting | Congress Abstract |
| Augustin  (2023) | Effects of a Mobile-Based Intervention for Parents of Children With Crying, Sleeping, and Feeding Problems: Randomized Controlled Trial | Different population |
| Flynn  (2023) | Smartphone-Based Video Antenatal Preterm Birth Education: The Preemie Prep for Parents Randomized Clinical Trial | Different population |
| Garfield  (2022) | A Mobile Health Intervention to Support Parenting Self-Efficacy in the Neonatal Intensive Care Unit from Admission to Home | Different study design |
| McKechnie  (2023) | An mHealth, patient engagement approach to understand and address parents' mental health and caregiving needs after prenatal diagnosis of critical congenital heart disease | Different study design |
| Olivia  (2019) | Smartphone-based prenatal education for parents with preterm birth risk factors | Different study design |
| Robinson  (2016) | Using telemedicine in the care of newborn infants after discharge from a neonatal intensive care unit reduced the need of hospital visits | Different study design |
| Hoodbhoy  (2021) | Role of community engagement in maternal health in rural Pakistan: Findings from the CLIP randomized trial | Different outcome |
| Gün  (2022) | A creative and practical approach to postpartum discharge education: Pecha Kucha training via smart phone | Different population |
| Jane Fisher (2016) | A new way of thinking about prevention of postnatal common mental disorders among primiparous women: evidence from a cRCT of a gender-informed psychoeducational program for couples. International Marcé Society Biennial Scientific Conference | Congress Abstract |
| Heather Rowe (2018) | Online and mobile psychoeducation initiatives to prevent postnatal mental health problems: research and evaluation. International Marcé Society Biennial  Scientific Conference | Congress Abstract |
| Shorey  (2019) | Effectiveness of a Technology-Based Supportive Educational Parenting Program on Parental Outcomes (Part 1): Randomized Controlled Trial | Different intervention |
| Abbass-Dick J (2019) | The comparison of access to an eHealth resource to current practice on mother and co-parent teamwork and breastfeeding rates: A randomized controlled trial | Different intervention |
