# Supplementary material for: Effects of perinatal mobile apps for couples on psychosocial and parenting outcomes: A systematic review and meta-analysis
Source: PLOS Ment Health. 2025 Oct 8;2(10):e0000432. doi: 10.1371/journal.pmen.0000432 (PMC12798352; doi:10.1371/journal.pmen.0000432)
Supplement: Attachment — Submitted filename: Response to Reviewers.docx [file pmen.0000432.s008.docx]

**Response to Editor and Reviewers – Manuscript ID: PMEN-D-25-00177**

**Dear Editor,**

We sincerely thank you and the reviewers for the time and effort dedicated to the evaluation of our manuscript entitled *“*Effects of perinatal mobile apps for couples on psychosocial and parenting outcomes: A systematic review and meta-analysis*”* (Manuscript ID: PMEN-D-25-00177). We are grateful for the insightful and constructive comments, which have helped us improve the quality and clarity of our work.

Please find below a point-by-point response to each of the comments raised by the reviewers and the editorial team. We have carefully revised the manuscript accordingly and clearly indicated all changes using tracked edits (or highlighted text, as preferred).

**Journal Requirements:**

*1. Please amend your detailed Financial Disclosure statement. This is published with the article. It must therefore be completed in full sentences and contain the exact wording you wish to be published.*

*i. Please clarify all sources of funding (financial or material support) for your study. List the grants (with grant number) or organizations (with url) that supported your study, including funding received from your institution.
ii. State the initials, alongside each funding source, of each author to receive each grant.
iii. State what role the funders took in the study. If the funders had no role in your study, please state: “The funders had no role in study design, data collection and analysis, decision to publish, or preparation of the manuscript.”
iv. If any authors received a salary from any of your funders, please state which authors and which funders.*

**Response:**
Thank you for your comment. We confirm that the authors received no specific funding for this work. Therefore:

i. There were no grants, institutional support, or external funding to list.
ii. No authors received any grants.
iii. No funders were involved in study design, data collection and analysis, decision to publish, or preparation of the manuscript.
iv. No author received a salary from any funder.

The revised statement now reads:

“The authors received no specific funding for this work.”

*2.* *We noticed that you used “unpublished” in the manuscript. We do not allow these references, as the PLOS data access policy requires that all data be either published with the manuscript or made available in a publicly accessible database. Please amend the supplementary material to include the referenced data or remove the references.*

**Response:** Thank you for your comment. We apologize for the confusion. In the manuscript, our use of the term “unpublished” was not intended to refer to a specific data source or reference, but rather to acknowledge the possible existence of studies that might not have been indexed or publicly available. To avoid misinterpretation and to comply with the journal’s data access policy, we have rephrased the sentence in the Limitations section as follows:

“Despite a comprehensive search across multiple databases, some relevant studies may have been missed due to lack of indexing or limited accessibility.”

This change has been made in the revised manuscript.

*3. As required by our policy on Data Availability, please ensure your manuscript or supplementary information includes the following:*

*A numbered table of all studies identified in the literature search, including those that were excluded from the analyses.*

*For every excluded study, the table should list the reason(s) for exclusion.*

*If any of the included studies are unpublished, include a link (URL) to the primary source or detailed information about how the content can be accessed.*

*A table of all data extracted from the primary research sources for the systematic review and/or meta-analysis. The table must include the following information for each study:*

*Name of data extractors and date of data extraction*

*Confirmation that the study was eligible to be included in the review.*

*All data extracted from each study for the reported systematic review and/or meta-analysis that would be needed to replicate your analyses.*

*If data or supporting information were obtained from another source (e.g. correspondence with the author of the original research article), please provide the source of data and dates on which the data/information were obtained by your research group.*

*If applicable for your analysis, a table showing the completed risk of bias and quality/certainty assessments for each study or outcome.  Please ensure this is provided for each domain or parameter assessed. For example, if you used the Cochrane risk-of-bias tool for randomized trials, provide answers to each of the signalling questions for each study. If you used GRADE to assess certainty of evidence, provide judgements about each of the quality of evidence factor. This should be provided for each outcome.*

*An explanation of how missing data were handled.*

*This information can be included in the main text, supplementary information, or relevant data repository. Please note that providing these underlying data is a requirement for publication in this journal, and if these data are not provided your manuscript might be rejected.*

**Response:** We appreciate the reviewer’s reminder regarding compliance with PLOS Mental Health’s data availability policy. We confirm that all required information is now clearly presented in the manuscript and/or supplementary materials, as detailed below:

A. Numbered table of all studies identified in the literature search, including excluded studies

- All studies identified through the literature search, including those excluded after full-text review, are listed in:
  - Table 1: *Study characteristics* – includes included studies.
  - Table 2: *Summary of Findings Table* – presents results for included studies.
  - S2 Table: *Excluded studies reviewed at full-text stage* – provides a list of excluded studies along with the reason(s) for exclusion.

B. Inclusion of unpublished studies

- We confirm that no unpublished studies were included in this review. All included studies were obtained from indexed and publicly accessible sources. No data from inaccessible or non-public sources were used.

C. Data extraction table including required metadata

- The complete data extraction table is provided in the supplementary file titled “S3 Table. Data extraction sheet for included studies”, which contains:
  - Names of data extractors and dates of data extraction.
  - Confirmation of study eligibility.
  - All data extracted from each study needed to replicate the analyses.

D. Data obtained via correspondence or other sources

- All data were primarily obtained from published articles.
- One clarification and additional data request was sent to Dr. Shefaly Shorey (nurssh@nus.edu.sg) regarding Shorey 2017, and the data were received on February 24, 2025.
- For Garfield FC 2016, we requested additional PSOC data but did not receive a response. Consequently, the PSOC mean score was extracted from the figure using R software (version 4.3.2), as described below (see S2 Appendix for the R script used).

E. Risk of bias and GRADE certainty assessment tables

- Risk of bias was assessed using the Cochrane Risk of Bias Tool (RoB 1.0). Detailed assessments for each domain are provided in Figure 2 of the manuscript.
- Certainty of the evidence was assessed using the GRADE approach across five domains, and judgments are provided for each outcome in Table 2: Summary of Findings.

F. Handling of missing data

- No missing data were reported in the included studies. However, for Garfield FC 2016, PSOC mean scores were not presented in tables or text. These values were extracted from the graphical representation using R software (v4.3.2). The png, graphics, and locator() functions were used to extract y-axis values, as is commonly done in systematic reviews when data are only reported in figures (see S2 Appendix for the R script).

*4. In the online submission form, you indicated that “The underlying data for this study are available in the supplementary information of the manuscript. Interested parties can request the data directly from the corresponding author via email. Additionally, the protocol for this study is registered in PROSPERO with the identifier CRD42024578397, accessible at the following link: (*[*https://www.crd.york.ac.uk/PROSPERO/)*](https://www.crd.york.ac.uk/PROSPERO/))*”.*

*All PLOS journals now require all data underlying the findings described in their manuscript to be freely available to other researchers, either
1. In a public repository,
2. Within the manuscript itself, or
3. Uploaded as supplementary information.*

*This policy applies to all data except where public deposition would breach compliance with the protocol approved by your research ethics board. If your data cannot be made publicly available for ethical or legal reasons (e.g., public availability would compromise patient privacy), please explain your reasons by return email and your exemption request will be escalated to the editor for approval. Your exemption request will be handled independently and will not hold up the peer review process, but will need to be resolved should your manuscript be accepted for publication. One of the Editorial team will then be in touch if there are any issues.*

**Response:**
Thank you for your clarification regarding PLOS’s data availability policy. In compliance with this requirement, we have now included a new supplementary file titled **S3 Table. Data extraction sheet for included studies**, containing the data underlying our systematic review and meta-analysis. This table provides detailed information on study characteristics, outcome measures, and other extracted variables.

We confirm that all data underlying the findings described in the manuscript are now publicly available within the supplementary materials, both in the submission system and in the manuscript. Additionally, the study protocol is registered and publicly accessible in PROSPERO (ID: CRD42024578397):<https://www.crd.york.ac.uk/PROSPERO/> and has also been provided as supplementary material under the title **S1 Protocol. PROSPERO**.

*5. Some material included in your submission may be copyrighted. According to PLOS’s copyright policy, authors who use figures or other material (e.g., graphics, clipart, maps) from another author or copyright holder must demonstrate or obtain permission to publish this material under the Creative Commons Attribution 4.0 International (CC BY 4.0) License used by PLOS journals. Please closely review the details of PLOS’s copyright requirements here: PLOS Licenses and Copyright. If you need to request permissions from a copyright holder, you may use PLOS's Copyright Content Permission form.*

*Please respond directly to this email or email the journal office and provide any known details concerning your material's license terms and permissions required for reuse, even if you have not yet obtained copyright permissions or are unsure of your material's copyright compatibility.*

**Response:** We appreciate the thorough editorial review and the opportunity to clarify this point. All figures, including the forest plots and the PRISMA flow diagram, were created by the authors using data extracted during our systematic review. Forest plots were generated using RevMan, and the PRISMA flowchart was built using the official PRISMA template with our own study data. Therefore, no third-party copyrighted content has been included, and no copyright permissions are required.

***Potential Copyright Issues:***

*We do not publish any copyright or trademark symbols that usually accompany proprietary names, eg (R), (C), or TM (e.g. next to drug or reagent names). Therefore please remove all instances of trademark/copyright symbols throughout the text, including Rayyan® on page 5.*

*If the reviewer comments include a recommendation to cite specific previously published works, please review and evaluate these publications to determine whether they are relevant and should be cited. There is no requirement to cite these works unless the editor has indicated otherwise.

Please review your reference list to ensure that it is complete and correct. If you have cited papers that have been retracted, please include the rationale for doing so in the manuscript text, or remove these references and replace them with relevant current references. Any changes to the reference list should be mentioned in the rebuttal letter that accompanies your revised manuscript. If you need to cite a retracted article, indicate the article’s retracted status in the References list and also include a citation and full reference for the retraction notice.*

**Response:**

We thank the editor for the helpful comments and guidance regarding copyright and reference standards.

We have removed all trademark and copyright symbols from the manuscript text, including the symbol previously used with Rayyan on page 5.

We have also carefully reviewed our reference list to ensure it is complete and accurate. No retracted articles have been cited in our manuscript. Therefore, no additional justification or changes were required in this regard.

We confirm that the manuscript now fully complies with the editorial requirements outlined.

**Comments to the Author**
1. Does this manuscript meet PLOS Mental Health’s [publication criteria](https://track.editorialmanager.com/CL0/https:%2F%2Fjournals.plos.org%2Fmentalhealth%2Fs%2Fcriteria-for-publication/1/010f0198371e17ea-0a94b36a-04b4-4c1e-984d-cd7459e742e6-000000/lS8n7yQtQgUrbHAKSWueMuFDF9Z5aCf4Q_lfJjjfULM=221)? Is the manuscript technically sound, and do the data support the conclusions? The manuscript must describe methodologically and ethically rigorous research with conclusions that are appropriately drawn based on the data presented.

Reviewer #1: Yes

Reviewer #2: Yes

2. Has the statistical analysis been performed appropriately and rigorously?

Reviewer #1: Yes

Reviewer #2: Yes

3. Have the authors made all data underlying the findings in their manuscript fully available (please refer to the Data Availability Statement at the start of the manuscript PDF file)?
The [PLOS Data policy](https://track.editorialmanager.com/CL0/https:%2F%2Fjournals.plos.org%2Fmentalhealth%2Fs%2Fdata-availability/1/010f0198371e17ea-0a94b36a-04b4-4c1e-984d-cd7459e742e6-000000/yOlypKELJWe2VgJ5hco5IWtpzpjehekAYhLCgFiHBys=221) requires authors to make all data underlying the findings described in their manuscript fully available without restriction, with rare exception. The data should be provided as part of the manuscript or its supporting information, or deposited to a public repository. For example, in addition to summary statistics, the data points behind means, medians and variance measures should be available. If there are restrictions on publicly sharing data—e.g. participant privacy or use of data from a third party—those must be specified.

Reviewer #1: Yes

Reviewer #2: Yes

4. Is the manuscript presented in an intelligible fashion and written in standard English?
PLOS Mental Health does not copyedit accepted manuscripts, so the language in submitted articles must be clear, correct, and unambiguous. Any typographical or grammatical errors should be corrected at revision, so please note any specific errors here.

Reviewer #1: Yes

Reviewer #2: Yes

**5. Review Comments to the Author**

**Reviewer #1 – Methods:** *“Revise the full-form of PROSPERO (International prospective register of systematic reviews)”*

**Response:**
We appreciate the reviewer’s suggestion. We have now revised the manuscript to include the full name of PROSPERO when first mentioned. Specifically, the sentence in the Methods section now reads:

“Our study protocol was registered in the **International Prospective Register of Systematic Reviews (PROSPERO)** under the identifier CRD42024578397, as detailed in S1 Protocol (https://www.crd.york.ac.uk/PROSPERO/).”

**Reviewer #1 – Methods:** *“State research question in 'Research question and eligibility criteria' section.”*

**Response:**
Thank you for your helpful comment. In response, we have now explicitly stated the research question at the beginning of the *Research question and eligibility criteria* section to improve clarity and alignment with systematic review reporting standards. The revised paragraph now begins with:

“Among couples and parents in the perinatal period, what is the effect of mobile application–based interventions, compared to standard care or no intervention, on psychosocial and parenting-related outcomes? To address this question, we included randomized controlled trials (RCTs) involving couples and parents during the perinatal period, excluding studies that focused exclusively on mothers.”

This addition improves transparency regarding the scope and focus of our review.

**Reviewer #1 – Methods:** *“Even though you have a separate supplementary file for search strategies utilized for each database, briefly describe major search terms in the 'search strategy' section.”*

**Response:**
Thank you for this useful suggestion. In response, we have revised the *Information sources and search strategy* section to include a brief description of the major search terms used. The section now reads:

“The search strategy comprised four main conceptual blocks: (1) population-related terms (e.g., parents, fathers, couples, pregnant women); (2) intervention terms related to mobile health (e.g., mHealth, smartphone, app, telemedicine); (3) outcomes related to mental health and parenting (e.g., depression, anxiety, bonding, self-efficacy); and (4) study design filters for randomized controlled trials. Detailed search strategies for each database are provided in Supplementary Table S1.”

**Reviewer #1 – Methods:** *“Can you give a bit more information about standardized data extraction form? Was there any specific form or did you make form in Excel?”*

**Response:**
 Thank you for your comment. We created a standardized data extraction form in Microsoft Excel specifically for this review. The form was developed by our review team to ensure consistency in data collection. As indicated in the *Methods – Variables* section of the manuscript, for interventions involving perinatal mobile applications, data were collected following the Template for Intervention Description and Replication (TIDieR) checklist, ensuring comprehensive and structured reporting of intervention characteristics.

The Excel form included the following main sections:

1. **General study information:** first author, study name, publication year, study year, country, study design, and funding.
2. **Population characteristics:** type of participants (e.g., couples, only fathers), inclusion/exclusion criteria for couples, parents, and newborns, age, sex, education, and other baseline characteristics.
3. **Intervention characteristics:** for each study arm – name, type, format (virtual or in-person), frequency/duration, provider, adaptations, adherence, and additional components.
4. **Subgroup analyses:** if applicable.
5. **Outcomes:**
   - *Dichotomous outcomes*: definition, follow-up time, events per group, effect measure (e.g., OR, RR), 95% CI, and comments.
   - *Continuous outcomes*: definition, follow-up time, type of comparison (e.g., difference-in-differences), mean/SD per group, number of participants, coefficient, and 95% CI.

**Reviewer #1 – Methods:** *Risk of bias assessment: Cochrane risk-of-bias tool for randomized trials (RoB 2) is the recommended tool to assess the risk of bias in RCTs. Why did you use RoB 1.0?*

**Response:**
We appreciate the reviewer’s suggestion to use the updated version of the Cochrane Risk of Bias tool. However, we opted to use the original Cochrane RoB 1.0 tool because, although RoB 2.0 provides a more detailed and structured framework, its application has been reported to be complex and may lead to reduced interrater reliability—even among experienced reviewers (Minozzi et al., 2020). For this reason, many systematic review authors continue to use RoB 1.0, which we considered more feasible and appropriate for the scope and resources of our review.

**Reference:**
Minozzi S, Cinquini M, Gianola S, Gonzalez-Lorenzo M, Banzi R. The revised Cochrane risk of bias tool for randomized trials (RoB 2) showed low interrater reliability and challenges in its application. *J Clin Epidemiol*. 2020;126:37–44.

**Reviewer #1 – Methods:** *Certainty of the evidence assessment: State full form of STAI scale. Correct spelling of 'literature' before citation [26].*

**Response:**
Thank you for your observations. We have now stated the full name of the STAI scale as “State-Trait Anxiety Inventory (STAI)” at its first mention in the manuscript. Additionally, we have corrected the spelling of “literature” before citation [26].

**Reviewer #1 – Results:** *While there is a separate figure 1 for study selection. I would suggest authors to include a brief description of selection (e.g., how many duplicates removed, how many were screened for full-text, etc.)*

**Response:**
We appreciate the reviewer’s suggestion. In response, we have revised the Results section to include a brief description of the study selection process. Specifically, we now report the number of duplicates removed, the number of records screened, excluded, and assessed for eligibility. The updated paragraph also maintains reference to the full list of excluded full-text articles and includes the flow diagram citation in accordance with PLOS style. The revised text now reads:

“During the systematic database search, 3347 records were initially identified. After removing 1856 duplicates, 1491 titles and abstracts were screened, of which 1472 were excluded. A total of 19 full-text articles were assessed for eligibility, and four randomized controlled trials (RCTs) were ultimately included in the review [28–31]. No additional RCTs were identified through complementary search methods. A detailed list of excluded full-text articles is provided in S2 Table, and the study selection process is shown in (Fig 1).”

**Reviewer #1 – Results:** *I believe this review is focused on the effect of mobile app (app-based intervention), but the fourth study (Abbass-Dick 2019) is related to providing intervention through a website (eHealth Breastfeeding Website). Can you please explain how did this study meet your inclusion criteria?*

**Author response:**

Thank you sincerely for this thoughtful observation. In our initial review, we considered including the study by Abbass-Dick et al. (2019) because, although the intervention was delivered via a website, the **Supplemental File #5** (*“Thematic Analysis of Feedback Regarding eHealth Resource”*) included participant feedback explicitly mentioning the use of **mobile devices**. For instance, participants referred to: *"usability on mobile devices"*.
Additionally, the study’s exclusion criteria specified that women were excluded if they did not have access to the internet or a telephone, which implied the use of mobile-compatible technology. Based on this information, we initially interpreted the intervention as a mobile-accessible web-based resource, potentially meeting our inclusion criteria, which focused on mobile app-based interventions.

However, after re-examining the study in depth and discussing it with all co-authors, we recognize that the intervention was not a mobile application per se, but rather a website-based eHealth resource. To further clarify, we contacted the study’s corresponding author via email, inquiring whether the intervention involved a mobile app. However, we have not received a response to date.

Given the potential confusion this study may cause regarding the scope of our review—focused specifically on interventions delivered primarily via mobile apps—we have decided to exclude Abbass-Dick (2019) from our systematic review and meta-analysis. Accordingly, we have updated the manuscript in all relevant sections (PRISMA flowchart, results, discussion, tables, and figures). We have also recalculated the meta-analysis, and the main findings remain robust and largely unchanged.

We are grateful for this valuable comment, which helped us strengthen the methodological clarity and consistency of our review.

**Reviewer #1 – Results:** - In addition to describing what study used what scales (e.g., EDPS, PPSS), I suggest authors to provide appropriate reference for those scales.

**Response:**

We thank the reviewer for this suggestion. In response, we have revised the Results section to include the original references for each measurement scale mentioned (e.g., EPDS, PPSS, STAI), in addition to citing the studies from our review that used them. These citations have been added at the first mention of each scale in the Results section.

**Reviewer #1 – Results:** In Table 2, in the first column (outcomes), I suggest citing those studies which reported each outcome (e.g., cite 2 RCTs reporting postnatal depression).

**Response:**
Thank you for this helpful suggestion. We have revised Table 2 to include the specific studies that reported each outcome, as suggested. The studies are now cited in parentheses next to each outcome in the first column for greater transparency and clarity.

**Reviewer #1 – Results:** Include results of meta-analyses below each figure heading.
 **Response:**
Thank you for the suggestion. While we appreciate the intent to enhance clarity, we have opted not to include the meta-analysis results below the figure headings, as this format is not commonly used in systematic reviews and meta-analyses. Instead, the effect estimates, confidence intervals, and heterogeneity metrics are clearly reported within each forest plot and in the accompanying narrative text. This approach is consistent with the presentation style of other published meta-analyses (e.g., Chan & Chen, 2019; Zafra-Tanaka et al., 2019).

**References:**

- Chan KL, Chen M. *Effects of Social Media and Mobile Health Apps on Pregnancy Care: Meta-Analysis*. JMIR MHealth UHealth. 2019;7(1):e11836. doi:10.2196/11836
- Zafra-Tanaka JH, Pacheco-Barrios K, Tellez WA, Taype-Rondan A. *Effects of dog-assisted therapy in adults with dementia: a systematic review and meta-analysis*. BMC Psychiatry. 2019;19(1):41. doi:10.1186/s12888-019-2026-8

**Reviewer #1 – Strengths and Limitations:**
“In strengths, authors mentioned that search was conducted without language restrictions. Did this review find studies that were conducted in languages other than English?”

**Response:**
Thank you for this important observation. While our search strategy did not apply any language restrictions, we confirm that all studies ultimately included in the review were published in English. To address this concern, we have revised the strengths section accordingly to clarify this point. The revised sentence now reads:
“A comprehensive search was conducted without language restrictions, enhancing the representativeness of the existing literature. However, we did not find any studies published in languages other than English.”

**Reviewer #2: This article is remarkably precise and well-composed. I commend the authors for their meticulous attention to detail and clarity throughout the manuscript.** *I have one suggestion to further enhance the article: in certain paragraphs—such as the one cited below—it would be beneficial to include individual references for each key component or claim. Citing a single reference at the end of a paragraph may lead to ambiguity regarding the source of specific information. Providing direct citations for each element will strengthen the transparency and traceability of the evidence presented.
"we evaluated five key domains: risk of bias (using the Cochrane risk of bias tool), imprecision (considering whether confidence intervals included one or two MIDs), indirectness (by comparing our PICO question (population, intervention, comparison, outcome) with those of the included studies), inconsistency (assessing whether studies point results were distributed on one or both sides of an MID), and publication bias (examined in meta-analyses with at least 10 studies) [23]."*

**Response to Reviewer 2**

We sincerely appreciate your positive feedback and your valuable suggestion. To improve the clarity and traceability of the sources used in key methodological components, we have revised the sentence to explicitly mention the GRADE Handbook at the beginning of the paragraph. This change ensures that the origin of each domain evaluated is clear from the outset. The revised sentence now reads:

“To assess the certainty of the evidence, we followed the GRADE Handbook guidelines [23] and evaluated five key domains: risk of bias (using the Cochrane risk of bias tool), imprecision (considering whether confidence intervals included one or two MIDs), indirectness (by comparing our PICO question with those of the included studies), inconsistency (assessing whether studies’ point results were distributed on one or both sides of an MID), and publication bias (examined in meta-analyses with at least 10 studies).”

6. PLOS authors have the option to publish the peer review history of their article ([what does this mean?](https://track.editorialmanager.com/CL0/https:%2F%2Fjournals.plos.org%2Fmentalthealth%2Fs%2Feditorial-and-peer-review-process%23loc-peer-review-history/1/010f0198371e17ea-0a94b36a-04b4-4c1e-984d-cd7459e742e6-000000/2kWi6toOwE8y7_4U7qYRJaGjr1zsso8UanhF3Tw9DRI=221)). If published, this will include your full peer review and any attached files.
Do you want your identity to be public for this peer review? If you choose “no”, your identity will remain anonymous but your review may still be made public.
For information about this choice, including consent withdrawal, please see our [Privacy Policy](https://track.editorialmanager.com/CL0/https:%2F%2Fwww.plos.org%2Fprivacy-policy/1/010f0198371e17ea-0a94b36a-04b4-4c1e-984d-cd7459e742e6-000000/I9E-9GyiLbinMMKwqO17eiDpwR911C2PE5WsMHOft78=221).

Reviewer #1: No

Reviewer #2: Yes: Azam Aslani

**Final Statement:**

We sincerely thank the Editor and Reviewers for their valuable feedback and thoughtful comments. We have carefully addressed each point raised, both editorial and peer-review, and have revised the manuscript accordingly. We hope that the changes made are satisfactory and that the revised version meets your expectations and the journal’s standards.

Thank you again for the opportunity to improve our work.

Sincerely,

Alvaro Taype-Rondan, on behalf of all authors
